# Supplementary material for: Large-scale digital forensic investigation for Windows registry on Apache Spark
Source: PLoS One. 2022 Dec 7;17(12):e0267411. doi: 10.1371/journal.pone.0267411 (PMC9728846; doi:10.1371/journal.pone.0267411)
Supplement: Attachment — Submitted filename: Summary of revision_v1_hykwon.docx [file pone.0267411.s002.docx]

Response to Reviewers

We deeply appreciate the reviewer’s careful concerns. The following is the explanation of the revision. Please note that, to satisfy the reviewer’s concerns, some of the figure numbers, page numbers, and line numbers have been changed.

**Reviewer#1, Concern # 1:** The paper needs some citations:

- In the second paragraph of the introduction, the authors describe the information stored in the Windows registry. The authors should provide a citation for this information.
- In section 2.1 the authors mention RegEdit but do not cite it. They should cite it.
- In section 2.1, the authors mention that RegEdit can extract data into a text file and briefly describe its structure. The authors should cite this information.
- The authors mention through the paper “Apache Spark”, “HDFS”, and “RegEdit” but do not cite their occurrences. They should.
- The authors should cite “Google Cloud Platform”, “Apache Hadoop 2.10.0”, and “Apache Spark 2.4.7” in section 5.1.

**Author response:** Thanks for your careful reviews. We have added the appropriate citations for all the parts that you pointed out.

**Author action:** We updated the manuscript as follows.

- **Action #1**: In Section 1, we added citations.

**1 Introduction**

…

The Windows registry stores critical information, including user accounts and program locations executed, when the system is booting [2, 3]. …

**References**

[2] Kwon HY. Constructing a lightweight key-value store based on the windows native features. Applied Sciences. 2019;9(18):3801.

[3] Lee JH, Kwon HY. Redundancy Analysis and Elimination on Access Patterns of the Windows Applications Based on I/O Log Data. IEEE Access. 2020;8:40640–40655.

- **Action #2**: In Section 2.1, we added citations.

**2 Background**

**2.1 Windows Registry**

… In this study, we extract the registry data from a target Windows system using **Regedit**[7], which is the built-in registry editor supported in any version of Windows systems. … We note that we can apply the existing forensic methods for Windows registry into the exported text file because all the information in the original registry is maintained in the text file, including the hierarchies of registry keys, i.e., the relationship between registry keys and their subkeys[2].

**References**

[2] Kwon HY. Constructing a lightweight key-value store based on the windows native features. Applied Sciences. 2019;9(18):3801.

[7] How to open Registry Editor in Windows 10. Available online: https://support.microsoft. com/en-us/windows/how-to-open-registry-editor-in-windows-10-deab38e6-91d6-e0aa-4b7c-8878d9e07b11 (accessed on 04-Jan.-2022).

- **Action #3**: In Section 2.2, we added citations.

**2 Background**

**2.2 MapReduce and Apache Spark**

… Apache Spark is an open-source software platform that supports the MapReduce framework[9, 10]. Apache Spark works with underlying systems for distributed environments, i.e., HDFS [11], for storing and managing large-scale data files and YARN for managing resources in a distributed environment.

**References**

[9] Apache Spark. Available online: https://sparkapacheorg (accessed on 04-Jan-2022).

[10] Zaharia M, Xin RS, Wendell P, Das T, Armbrust M, Dave A, et al. Apache spark: a unified engine for big data processing. Communications of the ACM. 2016;59(11):56–65.

[11] Borthakur D, et al. HDFS architecture guide. Hadoop apache project. 2008;53(1-13):2.

- **Action #4**: In Section 5.1, we added citations.

**5 Performance Evaluation**

**5.1 Experimental Environments and Data Sets**

…

In this paper, we use Google Cloud Platform[42] to build an actual distributed environment where Apache Hadoop 2.10.0[43] and Apache Spark 2.4.7[44] are installed. …

**References**

[42] Google Cloud Overview. Available online: https://cloudgooglecom/docs/overview (accessed on 04-Jan-2022).

[43] Apache Hadoop 2.10.0. Available online: https://hadoopapacheorg/docs/r2100/ (accessed on 04-Jan-2022).

[44] Apache Spark 2.4.7. https://sparkapacheorg/docs/247/ (accessed on 04-Jan-2022).

**Reviewer#1, Concern # 2:** The Introduction does not properly convey the importance of the paper. The authors mention that “The existing approach analyzes Windows registry targeting on a single Windows system. On the other hand, in our framework, we extract the Windows registry from several Windows systems and transform and load them into the Hadoop distributed file system (HDFS) on a Hadoop cluster“. However, they do not mention the flaws of single system analysis or of existing analysis nor why it would be necessary or important to process registry information from several Windows systems. The authors should modify the introduction by explicitly stating the shortcomings of single system analysis and the advantages of using HDFS.

**Author response:** Thank you for helpful comments so as to improve the quality of the paper. We have explained the limitations of the existing approach and the strength of the proposed approach.

**Author action:** We updated the manuscript as follows.

**1 Introduction**

…

In this study, we devised distributed algorithms to analyze large-scale registry data collected from a number of Windows systems on the Apache Spark framework. Fig.2 shows the overall proposed framework compared to the existing forensic approach on the Windows registry. The existing approach analyzes Windows registry targeting on a single Windows system. However, it can perform forensic analysis only for a single registry and has a limitation to identify malicious entries in a registry based on the comparison with other multiple Windows registry repositories. On the other hand, in our framework, we extract the Windows registry from several Windows systems and transform and load them into the Hadoop distributed file system (HDFS) on a Hadoop cluster. Then, we perform forensic analysis on large-scale data files stored on HDFS. Therefore, we can compare multiple Windows registry repositories in a scalable way and identify malicious registry entries based on them.

**Reviewer#1, Concern # 3:** In the Related Work section the authors summarize existing relevant works on Windows Forensics, Big Data Forensics, and Digital Forensics using Apache Spark and MapReduce. The authors only mention how their research is different from existing approaches in section 3.2 at the start of the paragraph. This statement should be moved to the end of the section to convey to the reader that their work is different than the existing approaches. Similar for section 3.3 and 3.1. The authors should write a paragraph at the end of those sections illustrating how their research is different from the other literature in those sections.

**Author response:** As you suggested, we have summarized the results of comparison with the existing studies in each subsection of related work and consistently described each of them in the last paragraph of the subsection.

**Author action:** We updated the manuscript as follows.

- **Action #1** In Section 3.1, we summarized the existing studies and the strength of the proposed work compared to them.

**3 Related Work**

**3.1. Windows Forensics**

…

As described, the existing Windows forensic studies focused on the registry entries extracted from a single system. They usually focused on target Windows registries where it has been known that suspicious information is stored. On the other hand, in this study, we propose a scalable forensic analysis of Windows registry by introducing Spark-based forensic framework to compare the entire Windows registry repositories collected from multiple systems, focusing on the differential registry entries.

- **Action #2:** In Section 3.2, we summarized the main differences of existing studies compared to our proposed work.

**3 Related Work**

**3.2. Big Data Forensics**

…

In summary, to the best of our knowledge, there have been no research efforts that focus on forensic analysis on the Windows registry in distributed nodes to deal with large-scale data. Instead, as described, there have been previous studies on large-scale data except for the Windows registry. Furthermore, most studies have focused on the concept of big data forensics or the overall framework rather than specific algorithms.

- **Action #3:** In Section 3.3, we summarized the existing studies and the strength of the proposed work compared to them.

**3 Related Work**

**3.3. Digital Forensics using Apache Spark and MapReduce**

…

These existing studies have also performed digital forensics through distributed frameworks as the same as our study. However, they have not considered the forensic analysis of Windows registry based on distributed frameworks. In this study, we present Apache Spark-based forensic analysis for Windows registry and propose a scalable analysis methodology for dealing with multiple Windows registry repositories for the first time.

**Reviewer#1, Concern # 4:** In section 5.1 the authors state “we measure the performance of the algorithm for loading registry data into HDFS proposed in Section 4.3 using up to eight nodes”. How are they measuring the performance? What metrics are being used? The authors should state this information in this section for experiment replication purposes.

**Author response:** We explicitly explained the measurement for the performance as pointed out.

**Author action:** In Section 5.1, we explicitly stated “processing time” instead of “performance” for the measurement.

**5 Performance Evaluation**

**5.1. Experimental Environments and Data Sets**

… For the first effect, we measure the processing time of the algorithm for loading registry data into HDFS proposed in Section 4.3 using up to eight nodes. For the second effect, we measure the processing time of the three scenarios by changing the number of nodes from 1 to 8. For the third effect, we measure the processing time of the three scenarios by changing the number of CPUs from 1 to 8 on each node.

…

**Reviewer#1, Concern # 5:** In section 5.1 in the second paragraph, the authors state “We collected actual Windows registry data from four different systems running Windows operating systems”. The authors should define what the specifications of these “Windows operating systems” are along with what data these systems were running for experiment replication purposes.

**Author response:** Thank you for important reviews to be reproducible research. We explained how we can extract the registry from the system, which can be performed for all the Windows-installed systems regardless of the Windows versions. Hence, we note that the proposed approach can be applied into any Windows registry.

**Author action:** We explained how we can extract the registry from the Windows systems. We also added the detailed information of the Windows systems. Finally, we noted that our proposed framework can be applied into any Windows registry only if we extract the registry entries from the systems.

**5 Performance Evaluation**

**5.1. Experimental Environments and Data Sets**

…

Table 1 lists the details of the collected Windows registry data. We can extract all the registry entries for a system into a text file with a Windows built-in command “regedit /E”[2]. Hence, we can easily collect the registry entries from all the Windows-installed systems including servers, desktops, and mobile devices, regardless of the Windows versions. The size of Windows registry files collected from Windows 10 depends on the system, i.e., 240MB, 352MB, 413MB, and 556MB, respectively. The detailed system information that is extracted from the registry is described in the table. We note that our proposed framework can be applied into any Windows registry only if we extract the registry entries using the command above. Hence, we used four registries in the table as the examples to measure the performance of the proposed algorithms. We note that their sizes vary widely according to the systems where *Registry_1_* is the registry obtained immediately after the Windows system is installed. This implies that a lot of information is added and updated in the registry while the Windows operating system is running. In total, we collected 1.561 GB of Windows registry, where over 2 million registry keys are stored.

**Reviewer#1, Concern # 6:** In section 5.2.1 the authors state “Here, we measure the performance varying the number of nodes and the number of CPUs.”. How is the performance being measured in this section? The authors should state the units and how the data was acquired for experiment replication purposes.

**Author response:**

1. We explicitly explained the measurement of the performance as pointed out.
2. Regarding the used data sets, please refer to the response and action to **Concern # 5**.

**Author action:** In Section 5.2.1, we explicitly stated “processing time” instead of “performance” for the measurement.

**5 Performance Evaluation**

**5.2. Experimental Results**

**5.2.1. Loading Windows Registry into Hadoop Distributed File System**

Fig. 11 shows the processing time of the algorithm proposed for loading Windows registry into HDFS (See Section 4.3). Here, we measure the processing time varying the number of nodes and the number of CPUs.

…
